# Altered Static and Temporal Dynamic Amplitude of Low-Frequency Fluctuations in the Background Network During Working Memory States in Mild Cognitive Impairment

**DOI:** 10.3389/fnagi.2019.00152

**Published:** 2019-06-28

**Authors:** Pengyun Wang, Rui Li, Bei Liu, Cheng Wang, Zirui Huang, Rui Dai, Bogeng Song, Xiao Yuan, Jing Yu, Juan Li

**Affiliations:** ^1^CAS Key Laboratory of Mental Health, Center on Aging Psychology, Institute of Psychology, Chinese Academy of Sciences, Beijing, China; ^2^Department of Human Resources, Institute of Disaster Prevention, Beijing, China; ^3^Department of Psychology, Zhejiang Normal University, Jinhua, China; ^4^Department of Anesthesiology and Center for Consciousness Science, University of Michigan, Ann Arbor, MI, United States; ^5^State Key Laboratory of Brain and Cognitive Science, Institute of Biophysics, Chinese Academy of Sciences, Beijing, China; ^6^School of Psychology, Capital Normal University, Beijing, China; ^7^School of Sociology, China University of Political Science and Law, Beijing, China; ^8^Faculty of Psychology, Southwest University, Chongqing, China

**Keywords:** mild cognitive impairment, working memory, amplitude of low-frequency fluctuations, background network, temporal dynamics

## Abstract

Previous studies investigating working memory performance in patients with mild cognitive impairment (MCI) have mainly focused on the neural mechanisms of alterations in activation. To date, very few studies have investigated background network alterations in the working memory state. Therefore, the present study investigated the static and temporal dynamic changes in the background network in MCI patients during a working memory task. A hybrid delayed-match-to-sample task was used to examine working memory performance in MCI patients. Functional magnetic resonance imaging (fMRI) data were collected and the marker of amplitude of low-frequency fluctuations (ALFF) was used to investigate alterations in the background network. The present study demonstrated static and dynamic alterations of ALFF in MCI patients during working memory tasks, relative to the resting state. Traditional static analysis revealed that ALFF decreased in the right ventrolateral prefrontal cortex (VLPFC), right dorsolateral PFC (DLPFC), and left supplementary motor area for normal controls (NCs) in the working memory state. However, the same regions showed increased ALFF in MCI patients. Furthermore, relative to NCs, MCI patients demonstrated altered performance-related functional connectivity (FC) patterns, with the right VLPFC and right DLPFC as ROIs. In terms of temporal dynamic analysis, the present study found that in the working memory state dynamic ALFF of bilateral thalamus regions was increased in NCs but decreased in MCI patients. Additionally, MCI patients demonstrated altered performance-related coefficient of variation patterns; the regions in MCI patients were larger and more widely distributed in the parietal and temporal lobes, relative to NCs. This is the first study to examine static and temporal dynamic alterations of ALFF in the background network of MCI patients in working memory states. The results extend previous studies by providing a new perspective on the neural mechanisms of working memory deficits in MCI patients.

## Introduction

Mild cognitive impairment (MCI) is a syndrome where individuals display certain forms of cognitive dysfunction, but the ability to perform basic daily activities remains intact (Petersen, 2004). Working memory, delayed recall, and spatial memory rapidly decline in the 6 years following MCI diagnosis (Cloutier et al., 2015). Many studies have focused on the deficit in working memory displayed by MCI patients (Klekociuk and Summers, 2014; Kirova et al., 2015) and the corresponding neural mechanisms, including altered activation during working memory tasks (Bokde et al., 2010; Lou et al., 2015; Migo et al., 2015). More recently, several studies have investigated the background network in MCI patients during working memory tasks. For example, previous studies found altered local synchronization (indexed by regional homogeneity, ReHo; Wang et al., 2016) and distant synchronization (indexed by degree of centrality, DC; Wang et al., 2017) of the background network in MCI patients during a working memory task. Additionally, Lou et al. (2015) found that MCI patients showed increased background network efficiency, which might compensate for the decreased activity and maintain the working memory state. However, the methods used to characterize the background network during the working memory state in MCI patients are generally at a network level, rather than at an individual voxel level. Specifically, ReHo employs the Kendall’s coefficient of concordance to measure the coordination of activity between voxels within a region, reflecting intra-regional synchronization [i.e., functional connectivity (FC; Zang et al., 2004)]. Similarly, the DC reflects the properties of the whole brain connectivity network. For a binary graph, DC is the number of edges connecting to a node. In a previous study, we distinguished local connectivity and distant connectivity and found only distant synchronization of the background network was changed in MCI patients (Wang et al., 2017). Thus, whether the oscillation of an individual voxel in the background network is altered in MCI patients during a working memory task is still unknown.

As early as 1995, Biswal et al. (1995) demonstrated that spontaneous low-frequency oscillations (<0.08 Hz) in the human brain during the resting state are physiologically meaningful. Amplitude of low-frequency fluctuations (ALFF) measures the total power of oscillations within a specific frequency range during a given time course (Zang et al., 2007). ALFF reflects individual voxel characteristics rather than the properties of the brain network because ALFF is calculated in each single voxel without the consideration of relationships with any other voxels. ALFF is physiologically meaningful in reflecting the function of brain network for both healthy and pathological populations, and have been used in various resting state functional magnetic resonance imaging (fMRI) studies. For example, ALFF is higher and dominant in the regions of default mode network than in other regions (Yang et al., 2007; Zang et al., 2007; Zou et al., 2008). Moreover, ALFF is significantly higher in bilateral visual cortices when scans conducted with eyes open than eyes closed (Yang et al., 2007). These measures also have implications for pathological populations. Abnormalities have been shown in ALFF in a number of regions implicated in the disorders in children with attention deficit hyperactivity (Zang et al., 2007), patients with major depressive (Liu et al., 2013), patients with Mesial temporal lobe epilepsy (Zhang et al., 2010), and in MCI patients (Han et al., 2011). More importantly, ALFF in some brain regions showed transformational pattern as the disease burden got heavy [from healthy older adults, to older adults with subjective cognitive decline, to MCI patients, to Alzheimer’s disease (AD; Yang et al., 2018)]. In the present study, we used this index to investigate whether the oscillations of individual voxels in the background network were altered in MCI patients during a working memory task.

In addition, in conventional resting state studies, FC and other properties (such as ReHo, DC, and ALFF) are assumed to be temporally stationary during a typical resting fMRI session of approximately 5–10 min. Based on this assumption, these measures are typically calculated over the entire duration of the resting fMRI session. However, this assumption may underestimate the complex and dynamic changes in interaction patterns (Chen et al., 2017), which have been demonstrated to contain valuable information (Smith et al., 2012; Allen et al., 2014). Given the increasing evidence of dynamic FC during resting states and its importance for characterizing the brain’s intrinsic functional organization, some studies have explored the temporal dynamics of FC during resting state in MCI patients. These studies have revealed that by combining evidence from the dynamic FC with that from traditional static FC, the diagnostic accuracy for MCI patients can be significantly improved (Wee et al., 2016; Chen et al., 2017; Zhang et al., 2017). However, these studies focus on dynamic changes during the resting state. To date, there are no studies exploring the dynamic network during a working memory task in MCI patients. Therefore, using the index of ALFF, the present study investigated the temporal dynamic changes in oscillations in the background network of MCI patients during a working memory task.

## Materials and Methods

### Participants

Participants were recruited from a community-based screening data pool in Beijing (healthy older adults, *n* = 865; MCI, *n* = 115; Dementia, *n* = 21; Yu et al., 2012, 2014; Yin et al., 2015). All the participants were asked to complete a neuropsychological battery and clinical assessment. Subsequently, some of the participants were selected to participate in the neuroimaging investigations. Experienced psychiatrists performed clinical diagnoses based on the results of previous tests and MCI patients were diagnosed according to the diagnostic criteria for MCI (Petersen et al., 1999, 2001). The psychiatrists’ clinical experience and scores on the Montreal Cognitive Assessment (MoCA; Nasreddine et al., 2005), the Mini-Mental Status Examination (MMSE; Folstein et al., 1975), and the Clinical Dementia Rating (CDR; Morris, 1993) were also used for the clinical diagnoses. Seventeen MCI patients and 16 healthy, age-matched control subjects (normal control, NC) participated in this study. See Table 1 for participants’ demographics and performance of neuropsychological tests and working memory task, which were also presented in our previous study (Wang et al., 2016). This study was approved by the research ethics committees of the Institute of Psychology, Chinese Academy of Science (H11036). Written informed consent was obtained from each participant.

**Table 1:** Summary of participants’ demographic information and performance of neuropsychological tests and working memory task.

	NC	MCI	*p*-value
*N*	16	17	-
Sex (male/female)	8/8	9/8	0.866
Age (years)	68.56 ± 5.76	70.53 ± 4.54	0.283
Education (years)	11.75 ± 3.17	9.82 ± 4.63	0.176
Self-rating	25.25 ± 4.51	29.08 ± 6.13	0.063
anxiety scale
ADL	14.19 ± 0.54	15.15 ± 2.30	0.115
MMSE	28.25 ± 1.39	24.47 ± 3.88	<0.010
MoCA	26.19 ± 1.52	19.18 ± 4.45	<0.001
CDR	0	0.5	-
Accuracy	91.12 ± 4.64%	77.50 ± 17.98%	0.007
Response time	609.76 ± 56.09	692.86 ± 86.36	0.003
Working memory	1.51 ± 0.15 (×10^−3^)	1.16 ± 0.36 (×10^−3^)	0.001
performance

*Note: NC, normal control; MCI, mild cognitive impairment; ADL, the Activities of Daily Life; MMSE, the Mini-Mental Status Examination; MoCA, the Montreal Cognitive Assessment; CDR, the Clinical Dementia Rating. To account for the tradeoff between accuracy and response time, working memory performance was measured using response accuracy divided by response time*.

### Working Memory Task: A Hybrid Delayed-Match-to-Sample Task (DMST)

Participants performed a modified DMST working memory task within the fMRI scanner (Jiang et al., 2000; Guo et al., 2008) as described in our previous study (Wang et al., 2016). In brief, the working memory task consisted of 32 trials separated into four blocks of eight trials. On each trial, two sample objects with green borders were presented side by side on the screen. Participants were asked to remember these two objects within 3,500 ms. Following this, test objects were presented. The test objects were selected from two groups: matching targets (the same as the sample objects) or non-matching distracter objects (new objects which differed from the sample objects). Target objects were presented two, three, or four times, and distractor objects were presented two, three, or four times, up to a total of 12 (or 13) test objects per trial. Each test object was presented for 1,000 ms separated by jitters of 800/900/1,000/1,100/1,200 ms. All test objects were pseudo-randomized and counter-balanced. During the test, participants were instructed to determine whether each test object matched the previously presented sample objects by pressing one of two buttons using their left or right thumb. The hand used for indicating a matching outcome was counterbalanced among participants. To avoid the effect of visual processing capacity, scrambled picture blocks (using the actual objects images) were presented alternatively with the DMST test trials, providing a baseline for each participant. Each of the scrambled picture blocks consisted of five pictures (2,000 ms for each picture). Participants were instructed to press both buttons when they perceived the scrambled pictures. Note that half of the test objects had been studied prior to scanning, whilst the other half were novel; however, this was not the focus of the present study.

### Image Acquisition

Participants were scanned using a Siemens Trio 3.0 tesla scanner (Erlangen, Germany) at the Beijing MRI Center for Brain Research. Images collected included during resting state, T1-weighted structural images, and during the working memory task. Both the resting and working state images were collected using the following parameters: repetition time (TR) = 2,000 ms, time echo (TE) = 30 ms, flip angle = 90°, field of view (FOV) = 200 × 200 mm^2^, 33 axial slices, thickness = 3.0 mm, gap = 0.6 mm, acquisition matrix = 64 × 64, and in-plane resolution = 3.125 × 3.125. During resting and working memory states, 200 and 652 (four runs) functional volumes were collected, respectively. During the resting state scanning, participants were instructed to keep their eyes closed, and not to think of anything in particular. T1-weighted structural images were acquired with the following parameters: 176 slices, acquisition matrix = 256 × 256, voxel size = 1 × 1 × 1 mm^3^, TR = 1,900 ms, TE = 2.2 ms, and flip angle = 9°. Full details of the scanning parameters have been published previously (Yu et al., 2016).

### Behavioral Data Analysis

The response accuracy on the working memory task was calculated according to the total hit rate (correct target detection) minus the total false alarm rate (false report for distractors). Response times (RTs) were calculated as the mean response time for all test stimuli (targets and distractors). As described in our previous study (Wang et al., 2016), to account for the tradeoff between accuracy and response time, working memory performance was further measured using response accuracy divided by response time, which is the reciprocal of the “inverse efficiency score” previously reported (Kennett et al., 2001; Spence et al., 2001).

### Image Preprocessing

Functional MRI data were preprocessed using the Statistical Parametric Mapping program (SPM8^1^) and the toolbox for Data Processing and Analysis of Brain Imaging^2^ (Yan and Zang, 2010). To acquire equal volumes of resting and working memory states, the second run of task data were selected to compare with the resting state. The first nine volumes of the working memory task data were discarded to allow for equilibration of the magnetic field, and the first 46 volumes of resting state data were discarded accordingly. All 154 volumes of both resting and working memory states were corrected for intra-volume acquisition time differences between slices using Sinc interpolation. A high-pass filter (128 s cut-off period) was used to remove low-frequency confounds. Spatial smoothing was then performed using a 4 mm full-width at half-maximum (FWHM) Gaussian kernel. Full details of the parameters used in the pre-processing have been published previously (Wang et al., 2016; Yu et al., 2016).

It should be noted that, in order to make the resting and working memory states comparable, for the working memory run an additional variable of task condition was included e.g., targets and distracters present vs. absent trials; response vs. no response trials. The rationale for removing task-load effects has been discussed previously (Jones et al., 2010; Gordon et al., 2012). By including this pre-processing step, the resting and task data differed only in the subjects’ cognitive state.

### Static ALFF

A whole-brain wise ALFF was calculated for each subject using the DPABI program. In brief, after pre-processing, the time series for each voxel was filtered (bandpass, 0.01–0.1 Hz) to remove the effects of very low-frequency drift and high-frequency noise (Biswal et al., 1995; Lowe et al., 1998). Subsequently, for each given voxel, the time series was converted to the frequency domain by a fast Fourier transform (parameters: taper percent = 0, length = shortest). The square root of the power spectrum was computed and averaged across a predefined frequency interval (0.01–0.1 Hz). This average square root value was defined as ALFF at the given voxel (Zang et al., 2007). For standardization purposes, all individual ALFFs were computed and standardized into ALFF *z*-values by subtracting the mean voxel-wise ALFF obtained for the entire brain (i.e., global ALFF), and then dividing by the standard deviation (SD; Zuo et al., 2013). This subject-wise ALFF normalization has been demonstrated to improve both normality and reliability across subjects (Zuo et al., 2013).

### Temporal Dynamic ALFF (TD-ALFF)

Dynamic ALFF was generated using sliding time window analysis by DPABI. First, rectangular windows (length of 32 TRs, overlapping by four TRs) were applied to BOLD signals to obtain a windowed time series. Rectangular windows (length of 64 and 128 TRs, overlapping by four TRs) were also applied to check that whether the patterns change with the length of rectangular windows. Second, the ALFF was calculated, as described above, within each window. Third, the mean, SD, and coefficient of variation (CV, SD/mean) maps were calculated across the time windows. The CV maps were then transformed to standardized z-scores, relative to the mean and SD across all voxels.

### Between-Group Comparisons of ALFF in the Resting State and the Background Network of the Working Memory State

To explore any interaction effects of group and cognitive state on ALFF, we performed a two-way repeated measures analysis of variance (ANOVA) using SPM8, with group (MCI vs. NC) as a between-subject factor and cognitive state (resting vs. working memory state) as a repeated measure. *Post hoc* two-sample *t*-tests were performed on clusters showing significant group × state interactions. The statistical threshold was set at *p* < 0.001 using the AlphaSim correction for multiple comparisons with a threshold of *p* < 0.01 at the voxel level (using DPABI). All coordinates are reported in the MNI format.

### Functional Connectivity (FC) Analysis

To examine the performance-related alterations in FC of the background network in MCI patients during a working memory task, we conducted a seed-based connectivity analysis using regions showing group × state interactions as seeds. First, for the background network during a working memory task for each individual, voxel-wise FC maps to a given seed were computed as maps of temporal correlation coefficients between the BOLD time course of each voxel and the averaged BOLD time course across voxels in the seed region. FC maps from individual subjects were then transformed using Fisher’s *z* transformation. Second, we calculated Pearson correlation coefficients (*p* < 0.05) to explore the relationship between the FC map and working memory performance in both the MCI and NC group. Bootstrap results were based on 1,000 bootstrap samples, and 95% confidence intervals are reported.

### Performance Related CV Maps During Working Memory State in MCI and NC

To explore which brain regions showed dynamic variation of ALFF related to working memory performance during the working memory state, Pearson correlation coefficients were calculated for both MCI and NC groups. The statistical threshold was set at *p* < 0.001 using the AlphaSim correction for multiple comparisons with a threshold of *p* < 0.05 at the voxel level.

## Results

### Alteration of Static ALFF Across Resting and Working Memory States in MCI and NC

**Figure 1:**
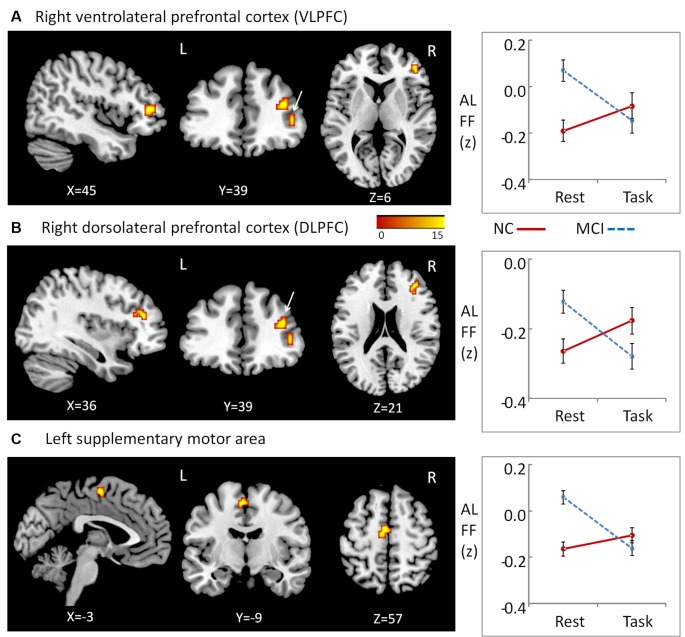
Regions showing Group × State interactions for amplitude of low-frequency fluctuations (ALFF) and the patterns of interaction. **(A)** Region in right ventrolateral prefrontal cortex (VLPFC), **(B)** region in right dorsolateral PFC (DLPFC), **(C)** regions in left supplementary motor area.

We observed significant interactions between group and state in the right ventrolateral prefrontal cortex (VLPFC; peak MNI coordinates: *x* = 45, *y* = 39, *z* = 6, cluster size = 10; average statistical coefficients of this region: *F*_(1,31)_ = 19.14, *p* < 0.001, $\eta_{\text{p}}^{2}$ = 0.38), right dorsolateral PFC (DLPFC; peak MNI coordinates: *x* = 36, *y* = 39, *z* = 21; cluster size = 15; average statistical coefficients of this region: *F*_(1,31)_ = 18.42, *p* < 0.001, $\eta_{\text{p}}^{2}$ = 0.37), and a region of the left supplementary motor area peak (MNI coordinates: *x* = −3, *y* = −9, *z* = 57; cluster size = 16; average statistical coefficients of this region: *F*_(1,31)_ = 26.71, *p* < 0.001, $\eta_{\text{p}}^{2}$ = 0.46; Figure 1). For all of the three clusters, further *post hoc* *t*-tests revealed that the value of ALFF was decreased in the resting state relative to the working memory state in MCI patients, but unchanged in NCs (Figure 1). Additionally, for all of the three clusters, *post hoc* *t*-tests showed that the value of ALFF was higher in MCI patients than in NCs during the resting state, while there was no difference between two groups during the working memory state.

Since these interaction effects were mainly due to abnormal hyperactivity in MCI patients during the resting state, the Pearson correlations between the brain ALFF activity and working memory performance in MCI patients was calculated to examine whether the hyperactivity was a compensatory process due to the reduced functionality of these brain regions. No significant correlation was found in MCI patients (*p* > 0.10).

### Performance-Related Functional Connectivity of Regions Showing Group × State Interactions During a Working Memory Task

To examine performance-related FC alterations of the background network in MCI patients during a working memory task, we conducted a seed-based connectivity analysis using regions that showed group × state interactions as seeds. Subsequently, we calculated Pearson correlation coefficients to explore the relationship between the FC map and working memory performance in both MCI and NC groups. The results demonstrate altered performance-related FC patterns in MCI patients when compared to NCs. In NC subjects, the working memory performance was related to FC between the right VLPFC and regions in the left inferior occipital gyrus (peak MNI coordinates: *x* = −45, *y* = −69, *z* = −12; cluster size = 257; *r* = 0.85, *p* < 0.001, Cohen’s *d* = 3.23), regions in the right inferior occipital gyrus (peak MNI coordinates: *x* = 30, *y* = −96, *z* = −9; cluster size = 402; *r* = 0.86, *p* < 0.001, Cohen’s *d* = 3.37), and the conjunction area of the limbic and occipital lobes (peak MNI coordinates: *x* = −18, *y* = −69, *z* = 6, cluster size = 170; *r* = 0.85, *p* < 0.001, Cohen’s *d* = 3.23). Also, working memory performance was related to FC between the right DLPFC and regions of the right middle occipital gyrus (peak MNI coordinates: *x* = 24, *y* = −90, *z* = 6; cluster size = 138; *r* = 0.87, *p* < 0.001, Cohen’s *d* = 3.53).

**Figure 2:**
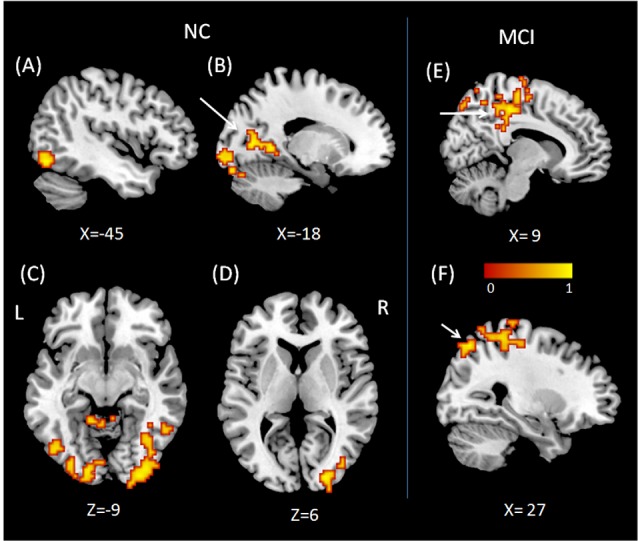
Regions showing performance related functional connectivity (FC) with right VLPFC **(A–C)** and right DLPFC **(D)** for normal control (NC). Regions showing performance related FC with right VLPFC **(E,F)** for mild cognitive impairment (MCI). **(A)** Region in left inferior occipital gyrus, **(B)** conjunction area of left limbic lobe and occipital lobe, **(C)** regions in right inferior occipital gyrus, **(D)** regions in right middle occipital gyrus, **(E)** right medial parietal cortex, **(F)** right superior parietal lobule.

In contrast, working memory performance in MCI patients was positively correlated with FC between the right VLPFC and regions of the right medial parietal cortex (peak MNI coordinates: *x* = 9, *y* = −30, *z* = 48; cluster size = 446; *r* = 0.86, *p* < 0.001, Cohen’s *d* = 3.37) and the right superior parietal lobule (peak MNI coordinates: *x* = 27, *y* = −69, *z* = 54; cluster size = 319; *r* = 0.91, *p* < 0.001, Cohen’s *d* = 4.39). No regions showed performance-related FC with the DLPFC in MCI patients. When regions in the left supplementary motor area were used as ROIs, we found no performance-related FC in either NCs or MCI patients (Figure 2).

### Alteration of Temporal Dynamic ALFF Across Resting and Working Memory States in MCI and NC Groups

**Figure 3:**
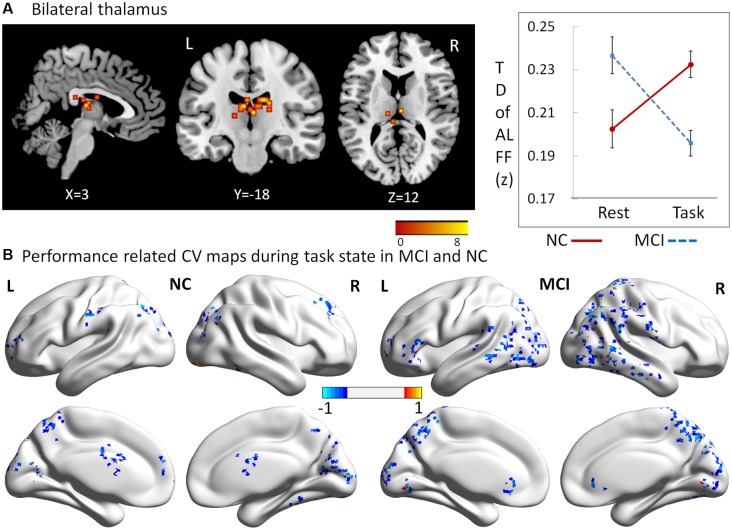
Regions showing alteration of temporal dynamic ALFF across resting and working memory states in MCI and NC groups. **(A)** Regions in bilateral thalamus, **(B)** performance related CV maps during the working memory state in MCI and NC.

To explore the interaction effects of group and cognitive state on CV of dynamic ALFF, we performed a two-way repeated ANOVA, with group (MCI vs. NC) as a between-subject factor and cognitive state (resting vs. working memory) as a repeated measure. Significant interactions between group and state were found in regions located in the bilateral thalamus (peak MNI coordinates: *x* = 3, *y* = −18, *z* = 12; cluster size = 52; average statistical coefficients of this region: *F*_(1,31)_ = 25.18, *p* < 0.001, $\eta_{\text{p}}^{2}$ = 0.45). Further *post hoc* *t*-tests revealed that the CV of dynamic ALFF was increased in the working memory state relative to the resting state in NCs but decreased in MCI patients (Figure 3A). It was noted that the patterns of the results were the same when used length of rectangular windows as 64 and 128 TRs.

### Performance Related CV Maps During the Working Memory State in MCI and NC

To explore which brain regions showed dynamic variation of ALFF in relation to the behavioral performance during the working memory state, Pearson correlation coefficients were calculated for both MCI and NC groups. Performance-related CV maps during the working memory state in MCI and NC groups are shown in Figure 3B. Two salient features were observed. First, the coefficients of correlation were negative in the majority of the regions, showing significant correlations with working memory performance in both groups. Second, relative to NCs, the regions in MCI patients were larger, and widely distributed in the parietal and temporal lobes.

## Discussion

The present study demonstrated alterations of static ALFF in MCI patients from the resting state to the working memory state. Specifically, the values of ALFF in the right VLPFC, right DLPFC, and left supplementary motor area decreased in the working memory state in MCI patients, but were unchanged in NCs. It should be noted that, compared to our previous study, which focused on the difference between MCI patients and NCs during the resting state, the main purpose of the current study was to uncover the alterations in MCI patients from resting state to working memory state. Therefore, the brain regions that showed interaction effects were the focus of this study. We found that relative to NCs, the brain ALFF activation in the right VLPFC, right DLPFC, and left supplementary motor area was decreased from the resting to the working memory state in MCI patients. These interaction effects were mainly due to abnormal hyperactivity in MCI patients during the resting state. In the resting state, two regions in the lateral PFC (in both the VLPFC and DLPFC) showed higher ALFF in MCI patients, compared to NCs. These results are in agreement with some previous studies but in disagreement with others. On the one hand, some previous studies have found the inverse pattern of results to the present study. Han et al. (2011) found that MCI patients had decreased ALFF/fALFF activity in several lateral prefrontal regions. Similarly, using independent component analysis (ICA), Sorg et al. (2007) also found that MCI patients had reduced spontaneous activity in the lateral prefrontal regions. On the other hand, however, other studies have demonstrated similar results to our experiments. Using ReHo, our previous study revealed that MCI patients had higher ReHo than NCs in the lateral prefrontal regions during the resting state (Wang et al., 2016). Using the index of ICA, Qi et al. (2010) also found that MCI patients had increased brain activity in lateral prefrontal regions, thought to be compensatory hyperactivity. Based on the current evidence, it is not certain whether these changes (decrease or increase) in MCI patients are damaging or compensatory. Cabeza et al. (2018) have suggested that for hyper activation to be considered compensative, it ought to be positively related with cognitive performance. No direct evidence of a correlation has been provided by previous studies and no significant correlation was found in either MCI or NC groups in the current study. An alternative explanation is that the changes represent a deficit of function. The DLPFC and VLPFC are part of the executive network, whereas the left supplementary motor area belongs to the motor network (Yeo et al., 2011). These networks are quieter during the resting state in contrast to the default mode network in normal adults (Yeo et al., 2011). Therefore, the hyperactivity of these networks in MCI patients in the present study is more likely to be a deficit of inhibitive function.

The next question relates to the FC between abnormal brain regions and other parts of the brain, and how this is related to the behavioral performance during a working memory task. Using FC analysis with right VLPFC and right DLPFC as ROIs, the present study found that MCI patients demonstrated altered performance-related FC patterns as compared to NCs. For NCs, the FC between VLPFC, DLPFC, and regions in the bilateral inferior occipital gyrus and limbic lobe might facilitate working memory performance. For MCI patients, those with higher FC between VLPFC and regions in the right medial parietal cortex and right superior parietal lobule showed better working memory performance.

The delayed-match-to-sample task requires participants to judge continuously whether the present picture is the same as the previous one. Thus, the task requires the use of the executive component in order to access images maintained in the “visuospatial sketchpad” (Baddeley, 1992). Therefore, the communication between the central executive system and the “visuospatial sketchpad” is critical for successful working memory performance. Regions of the PFC, including the DLPFC and VLPFC, have been suggested as key components of the central executive system (Nee et al., 2013; D’Esposito and Postle, 2015), whereas regions in the bilateral occipital lobe have been described as critical for visual image representation. For example, it has been found that short-term retention of complex visuospatial patterns (Christophel et al., 2012), objects, faces, houses, scenes, and body stimuli (Han et al., 2013; Lee et al., 2013; Sreenivasan et al., 2014), relies on the occipital cortex and other areas in the parietal and temporal cortices. The occipital cortex has also been implicated in processes related to reconstructed images and observed images (Ishai et al., 2000; Rissman and Wagner, 2012). Therefore, our finding, that the FC between the PFC and the occipital lobe was positively correlated with working memory performance in NCs, demonstrates that efficient information transfer between the central executive system and the visuospatial sketchpad in the background network is necessary for efficient working memory function. However, this critical neural pathway was not found in MCI patients. Instead, higher FC between the right VLPFC and regions in the right medial parietal cortex and the right superior parietal lobule facilitated working memory performance in MCI patients. This pathway has been considered as critical for the right frontoparietal network, which is important for working memory function, especially involving the executive component (Yeo et al., 2011). This is a more fundamental neural pathway for the performance of successful working memory. The two distinct neuronal pathways in MCI patients and NCs may suggest that for MCI patients, the fundamental pathway of working memory is impaired. Subsequently, the individual differences observed in working memory performance in MCI patients depend on the functionality of this pathway. For NCs, however, the fundamental neural structure for working memory is intact, thus their individual differences mainly result from the superior neural circuit, such as the pathway between the PFC and occipital areas. As the frontoparietal network is a fundamental pathway for working memory, we suggest that the hyperactivity observed in MCI patients is not compensatory, as suggested by Cabeza et al. (2018).

Recent studies have demonstrated that dynamic changes in neural interactions contain valuable information (Smith et al., 2012; Allen et al., 2014). To characterize the brain’s intrinsic functional organization more deeply, we explored the dynamic changes during resting and working memory states in MCI patients and NCs. We found that the dynamic ALFF of bilateral thalamus regions was increased in NCs but decreased in MCI patients during the working memory state, relative to resting state. These results provide further evidence that the thalamus plays a key role in working memory. Previous work has shown that working memory capacity and executive function share a common underlying executive attention component (McCabe et al., 2010). Additionally, long-range communication of information between brain regions also likely plays an important role in working memory function (Sauseng et al., 2005; Crespo-Garcia et al., 2013). For example, in a human MEG study, synchronized oscillations in the alpha, beta, and gamma bands were observed between frontoparietal and visual areas during the retention interval of a visual working memory task. These synchronized oscillations were memory load-dependent and correlated with an individual’s working memory capacity, suggesting a mechanism for effective communication between brain regions involved in the temporary maintenance of relevant visual information (Palva et al., 2010). Critically, the thalamus has been suggested to be not only key in attentional selection, but more generally in regulating information transmission across the cortex (Saalmann et al., 2012). In the present study, the dynamic ALFF in bilateral thalamus regions was increased from the resting to the working memory state in NCs, which suggests that the thalamus works as an executive attention component and an effective communication regulator during the working memory task. For MCI patients, however, this mechanism was inversed. The dynamic ALFF in these regions was decreased in the working memory state, which may result in a weakened ability of attentional selection and information regulation, resulting in an impairment of working memory function.

Next, we investigated in which regions the dynamic variation of ALFF was related to the behavioral performance during the working memory state. As outlined in the “Results” section, two salient features were observed in the performance-related CV maps. First, the coefficients of correlation were negative in the majority of the regions that showed significant correlations with working memory performance in both groups, which suggests the dynamic variation of ALFF in these regions reduced performance on the ongoing behavioral task. Second, MCI patients demonstrated altered performance-related CV patterns relative to NCs. The altered regions in MCI patients were larger, and widely distributed in the parietal and temporal lobes. Given the negative effect on the working memory task, these results indicate that MCI patients exhibit widespread and varied low-frequency fluctuations in the parietal and temporal lobes, which reduce their working memory function.

It has been found that some regions in frontal, parietal and temporal lobes can be modulated noninvasively to improve neural networks and eventually memory tasks (Wang et al., 2014; Koch et al., 2018). From a translation point of view, the altered brain regions in MCI patients found in the present study (such as VLPFC, DLPFC, and supplementary motor area) may be identified as target areas for that are precociously altered in the degenerative process leading to dementia.

One of the limitations of the present study was that no biomarker (such as cerebrospinal fluid, or amyloid positron-emission tomography scan) was included during the clinical diagnosis of MCI patients. Therefore it was impossible to identify subtypes of MCI patients according to the intrinsic pathological mechanism. Considering the high pathological heterogeneity of clinically diagnosed MCI patients, each subtypes may exhibit different patterns in the issues of the present study, which should be explore further in the following studies.

To the best of our knowledge, this is the first study to examine alterations of static and dynamic ALFF in the background network of MCI patients during working memory states. The results provide a new perspective regarding the neural mechanisms of working memory deficits in MCI patients and extend our knowledge of altered brain patterns in resting and task-evoked states.

## Ethics Statement

This study was approved by the research ethics committees of the Institute of Psychology, Chinese Academy of Science (H11036). Written informed consent was obtained from each participant.

## Author Contributions

PW conceived the idea, designed the study, analyzed and interpreted data, drafted part of the manuscript. RL, CW, RD, and ZH assisted to analyze and interpret the data. BL, BS, and XY assisted to analyze the data, make charts, and drafted part of the manuscript. JY carried out the experiment and drafted part of the manuscript. JL conceived the idea, designed the study, and participated in writing up and revising the manuscript.

## Conflict of Interest Statement

The authors declare that the research was conducted in the absence of any commercial or financial relationships that could be construed as a potential conflict of interest.

## Footnotes

^1^http://www.fil.ion.ucl.ac.uk/spm

^2^http://rfmri.org/dpabi
